# Pipeline for imaging, extraction, pre-processing, and processing of time-series hyperspectral data for discriminating drought stress origin in tomatoes

**DOI:** 10.1016/j.mex.2019.02.022

**Published:** 2019-02-26

**Authors:** Uroš Žibrat, Nik Susič, Matej Knapič, Saša Širca, Polona Strajnar, Jaka Razinger, Andrej Vončina, Gregor Urek, Barbara Gerič Stare

**Affiliations:** Agricultural Institute of Slovenia, Plant Protection Department, Hacquetova ulica 17, 1000 Ljubljana, Slovenia

**Keywords:** Hyperspectral image processing, Hyperspectral imaging, Remote sensing, PLS-DA, PLS-SVM, Drought stress, Root-knot nematode, *Meloidogyne*, Tomato, *Solanum lycopersicum*, Detection, Stress, Biotic, Abiotic

## Abstract

Crop infestation with root-knot nematodes (RKN) and water deficiency lead to similar visible symptoms in the plant canopy. Identification of biotic or abiotic stress origin is therefore a problem, and currently the only reliable methods for determination of RKN infestation are invasive and applicable only for point-searches. In this study the applicability of hyperspectral remote sensing for early identification of drought stress and RKN infestations in tomato plants was tested. A four-stage image and data management pipeline was established: (1) image acquisition, (2) data extraction, (3) pre-processing, and (4) processing.

• This pipeline reduces atmospheric impacts, facilitates data extraction (by using specially designed spectral libraries and supervised classification procedures), diminishes the impact of viewing geometry, and emphasized small spectral variations not apparent in the raw data.
• By combining partial least squares – discriminant analysis and support vector machines with time series analysis, we achieved up to 100% classification success when determining watering regime and infestation, and their severity.
• This pipeline could be at least partially automated, thus facilitating high throughput identification of stress origin in plants. Furthermore, the same pipeline could be applied to hyperspectral phenotyping procedures, which are gaining importance in breeding programs.

## Graphical abstract

**Figure:**
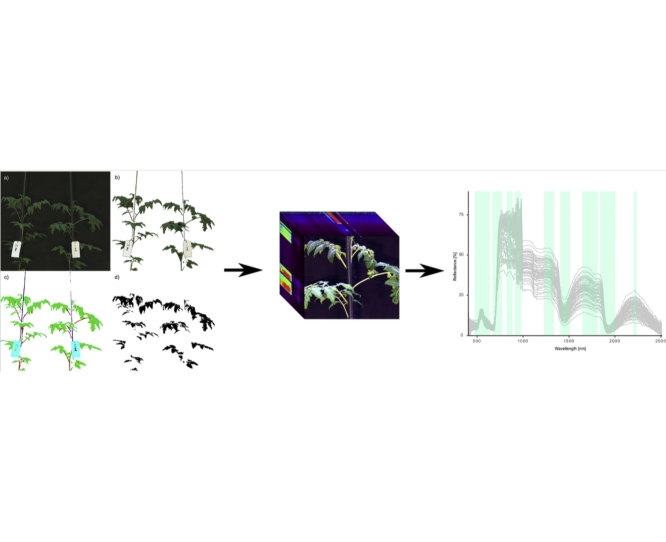

**Specifications Table**

**Table:** 

**Subject Area:**	*Agricultural and Biological Sciences*
**More specific subject area:**	*Precision agriculture and plant protection*
**Method name:**	*Hyperspectral image processing*
**Name and reference of original method:**	H. Huang, L. Liu, M. Ngadi, Recent developments in hyperspectral imaging for assessment of food quality and safety, Sensors. 14 (2014) 7248–7276. https://doi.org/doi:10.3390/s140407248.
**Resource availability:**	*IEnvi,*https://www.harrisgeospatial.com/SoftwareTechnology/ENVI.aspx *Unscrambler,*http://www.camo.com/rt/Products/Unscrambler/unscrambler.html *R,*https://www.r-project.org/

## Method details

### Background

Plant-parasitic nematodes have a major impact on global food production, with an annual loss of approximately $100 billion worldwide [1]. Root knot nematodes (RKN) of the genus *Meloidogyne* are one of the most important agricultural pests [[2], [3], [4]], accounting for approximately 5% of global crop losses [5]. The group of tropical RKNs can parasite a wide range of host plants, hundreds of agricultural crops belonging to monocotyledons, dicotyledons, including herbaceous and woody plants.

The nematodes actively enter growing plant roots and elicit development of feeding sites where visible root galls develop. The deformed root system thus leads to reduced uptake of water and nutrients, and overall weakening of the host plants [6]. Visible symptoms of infestation on photosynthetically active tissue are akin to those of water deficiency, making accurate identification of stress origin difficult. Furthermore, these symptoms develop during the last stages of nematode infestation, making early detection vitally important. Visually checking the root system for galls is currently the most reliable method of determining RKN infestation. However, this approach is invasive and useful only for point-searches on individual plants. Remote sensing applications enable non-invasive and early detection of drought stress, and its origin (biotic or abiotic). Moreover, detection can be performed on several plants at the same time, thus covering larger areas or more plants than with point-searches.

Hyperspectral imaging is a passive remote sensing method, which combines the benefits of two major techniques, imaging and spectroscopy [7]. These devices record image planes of the object under study, at different wavelengths, and superimposes these, forming a three-dimensional data cube, the hyperspectral image [8]. After radiometric corrections, reflectance can be computed. Analyses of leaf reflectance can provide physiological and morphological information about plants, such as leaf pigmentation and presence of stress [9].

The aim of this paper is to present a hyperspectral data processing pipeline, which can be used as-is or be adapted for other applications.

### Experimental design

Tomato (*S. lycopersicum*) hybrid ‘Horus H1’ (L’Ortolano, Italy) plants were divided into six groups each with seven biological replicates (a single plant per pot) (N = 42). Prior to germination seeds were sterilized using a 3% aqueous solution of sodium hypochlorite (NaOCL; Kemika, Croatia), and germinated in the dark on 1/3 potato dextrose agar (PDA; Biolife, Italy) for 6 days at T = 22 °C. Sprouted seeds without signs of bacterial or fungal infections were transferred to sterile plant substrate and grown in trays in a climate controlled environment for 16 days. Plants were planted in a mixture of 2 parts fine-grain (MP1/G), 2 parts coarse-grain (MP4) quartz sand (Termit, Slovenia), and 1 part fine peat substrate Potgrond P (Klasmann-Deilmann, Germany), with a final substrate density of 1.25 g/cm^3^. Plantlets were then transplanted into styrofoam multitrays and grown for 35 days, until the root system was well developed. Finally, 51-day-old plants were transplanted to 5 L polypropylene pots with a diameter of 25 cm, with the same substrate mixture. In three randomly selected pots temperature sensors iButton (Maxim, USA) were embedded in the substrate. Temperature measurements were used to follow the life cycle of nematode *Meloidogyne incognita* and predict the completion of the first life cycle, according to the model developed by Širca et al. [10].

**Fig. 1:**
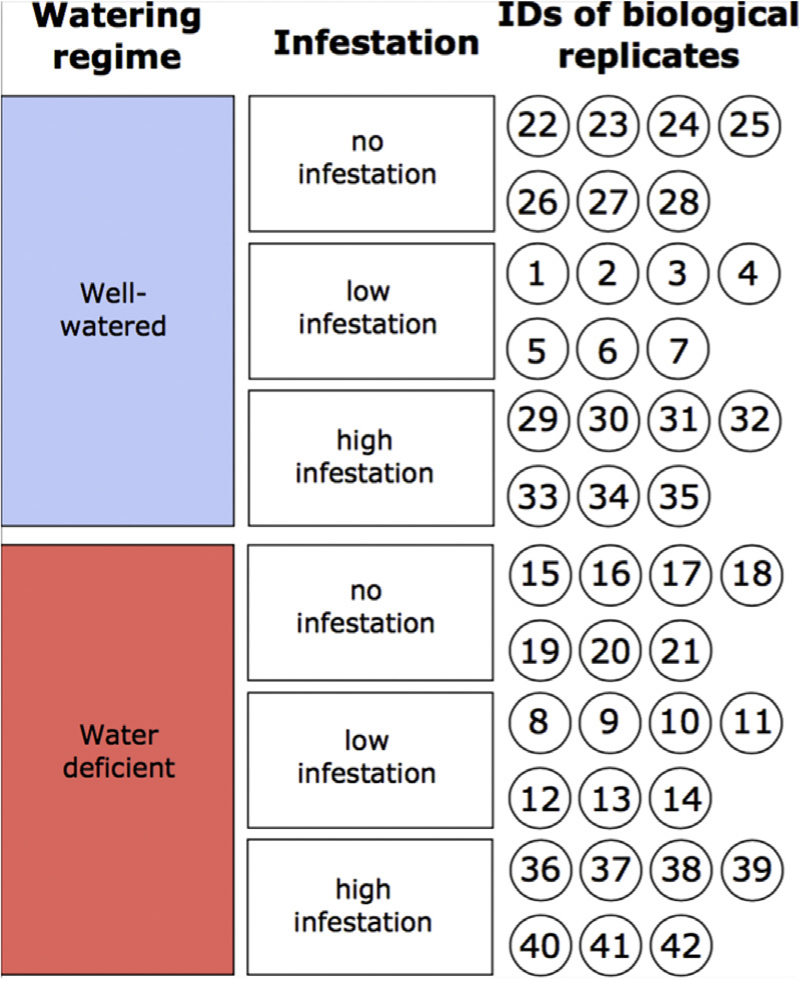
Setup of the experiment. Plants were randomly assigned to one of six treatment groups, with seven biological replicates each.

Plants were subjected to two sources of stress, abiotic and biotic. For the former, two watering regimes were used, to attain well-watered and water-deficient conditions, the latter eliciting chronic drought. Biotic stress was achieved by root-knot nematode infestation. Plants were subjected to three levels of initial nematode inoculum: (1) no infestation, (2) low infestation (15 × 10^3^ *M. incognita eggs*, equaling 3 eggs cm^−3^ substrate), and (3) high infestation (250 × 10^3^ eggs, equaling 50 eggs cm^−3^ substrate) (Fig. 1). Plants were watered daily with a three-component nutrient mixture Flora Series (General Hydroponics Europe, France) for hydroponics-based systems. The nutrient mixture was prepared by mixing three Flora Series (N-P-K) solutions: FloraGro 3-1-7, FloraMicro 5-0-1 and FloraBloom 0-5-4, according to the manufacturer’s guidelines with regard to the plant developmental stage. Drought conditions were initiated 8 days after inoculation (DAI) by irrigating plants with the lowest volume of nutrient solution sustaining turgor pressure. Well-watered plants were irrigated to substrate saturation. All plants received the same amount of nutrients, regardless of watering regime.

### Hyperspectral imaging and spectral acquisition

Tomatoes were scanned using two pushbroom imaging spectrometers; HySpex VNIR (spectral range 400–988 nm) and SWIR (spectral range 950–2500 nm) (Norsk Elektro Optikk AS, Norway). The cameras were mounted horizontally on a tripod coupled with a rotation stage, so the rotation speed was synchronized with the scanning cameras frame rate and field of view. The system was controlled by the data acquisition unit using HySpex GROUND software as supplied by the manufacturer. The imaging system setup included two calibrated halogen light sources with an even light intensity between 400 and 2500 nm, which were switched on 15 min prior to imaging in order to stabilize light source temperature drift and establish spatial lighting uniformity [11]. The cameras were positioned at 1.5 m from ground level and 3 m distance from the imaged tomato plants – the resulting field of view per image was 1 × 2.5 m. Using this arrangement, up to 3 plants could be imaged at the same time against a black background screen (reflectance <5%). Every image also included a calibrated diffuse white reference plate with 95% reflectance (SphereOptics, Germany). Hyperspectral imaging was performed at three sessions: (1) at 12 DAI (labelled S1), (2) 34 DAI (S2), and (3) 52 DAI (S3). The imaging sessions correspond to early stages of infestation (S1), middle of the nematode development cycle (S2), and completed first reproduction cycle (S3).

### Data pre-processing

**Fig. 2:**
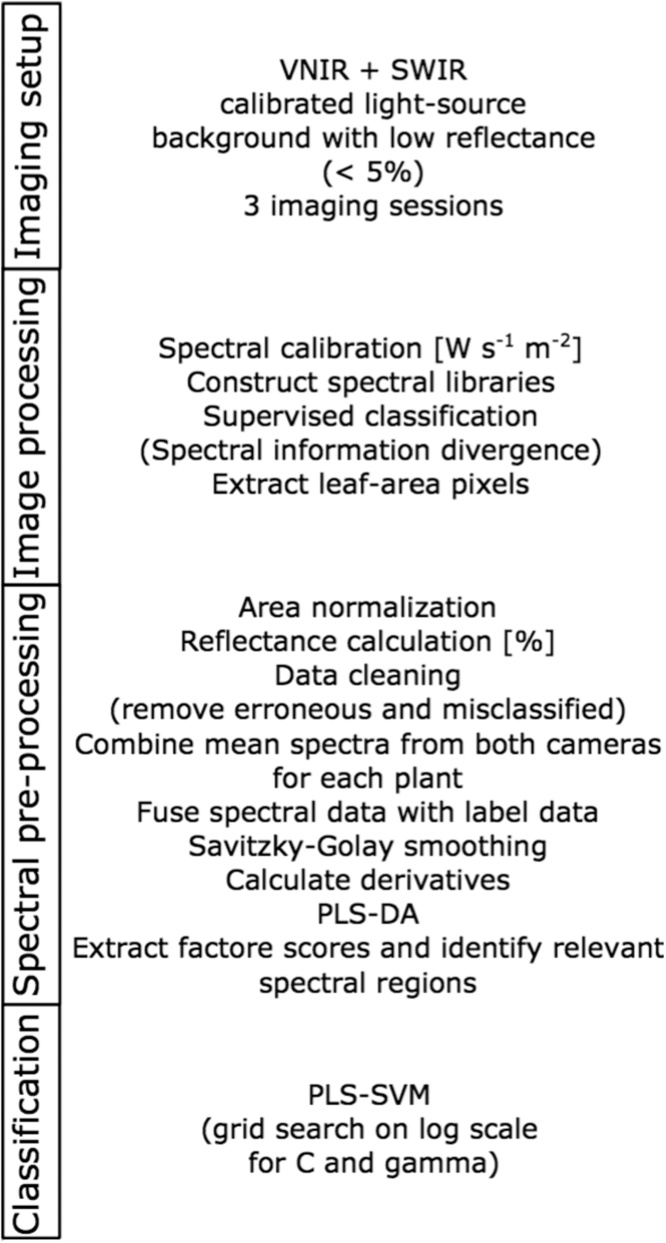
Image and data processing pipeline. The process was divided into four parts. The first three parts were applied to each plant in each imaging sessions, and classifications (part four) were performed for each imaging session and for all sessions combined.

**Fig. 3:**
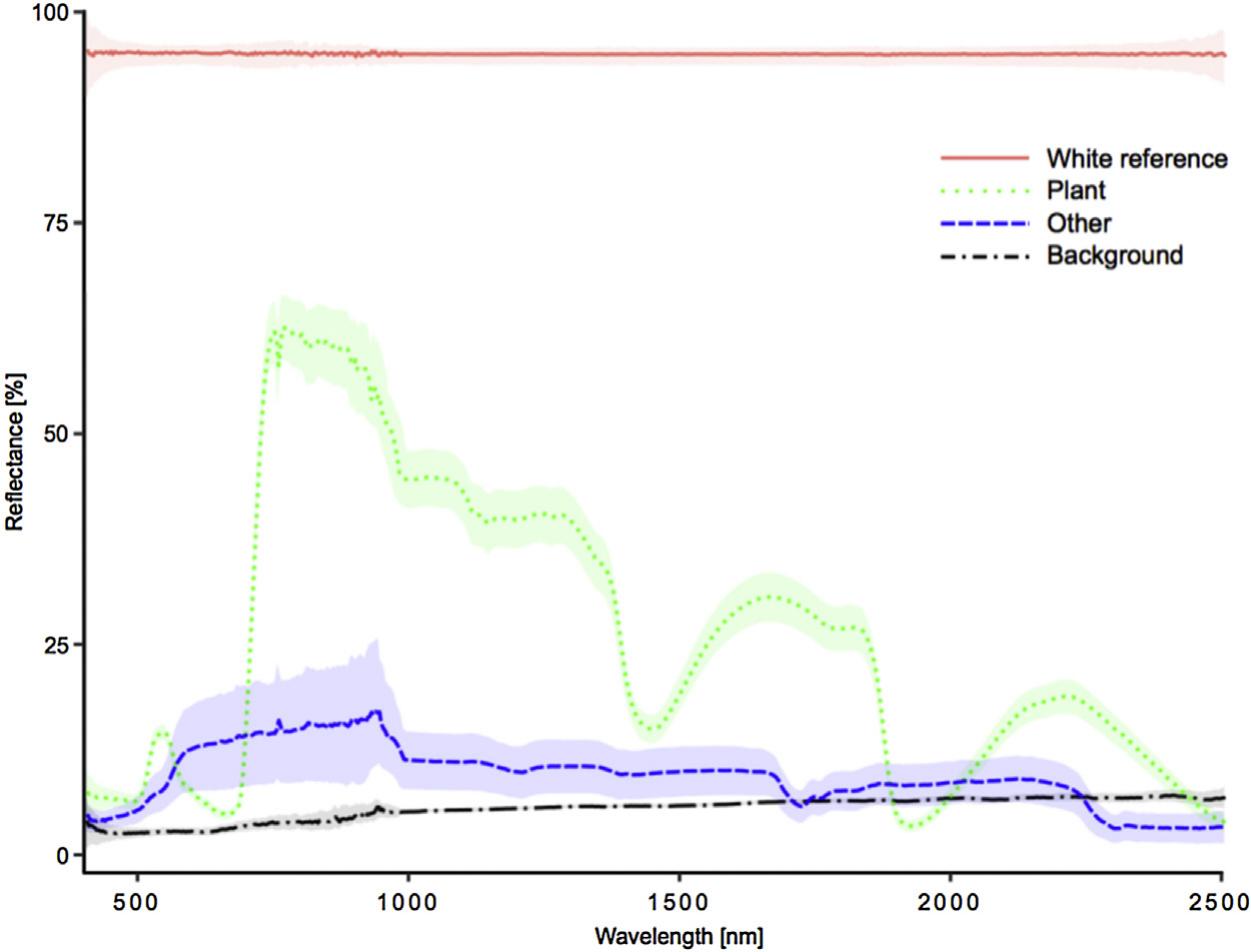
Spectral signatures of the four classes included in the spectral libraries. Lines denote mean spectra, and ribbons their according standard deviations.

**Fig. 4:**
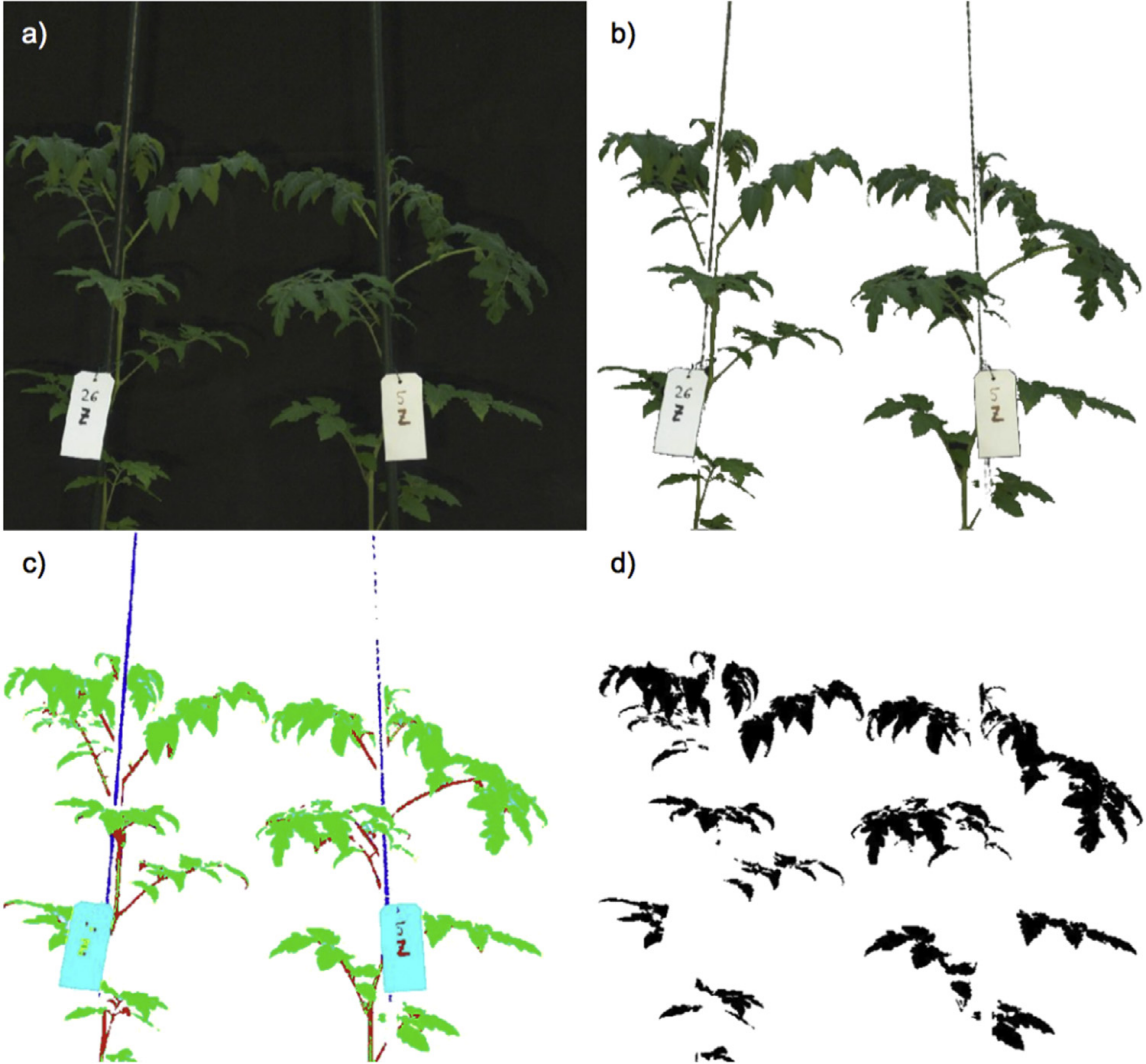
Extraction of leaf-area pixels: a) original hyperspectral image; b) background removal; c) supervised classification using spectral information divergence; and d) final mask used to extract leaf-area pixels.

Pre-processing was based on the guidelines by Huang et al. [8] and Shrestha et al. [12] (Fig. 2). All hyperspectral images were radiometrically calibrated to radiance units (W sr^−1^ m^−2^). We established spectral libraries with four classes: (1) plants, (2) background, (3) white reference, and (4) other. Members of the last class were pots, sticks, and other artificial materials, used for growing plants, as support or as markers. Each imaging session and camera had its own spectral library, hence six libraries were constructed (Fig. 3). These spectral libraries were used as endmember collections for supervised classification using spectral information divergence (SID) [13]. SID classification was performed on each image, in order to extract leaf-area pixels (Fig. 4). Classification success for identification of plants was 99.76%. Pixel values of each plant were normalised using area normalization, due to the variable geometry of imaged plants. Data from white reference plates was extracted using the same process (SID classification success 100%). Reflectance values for each band of each image pixel (R) were calculated as:

Ri = (I_i_ – D_i_)/((W_i_ – D_i_)/0.95),

where *I_i_* represents the reflected signal of the i-th band; *W_i_* is the reflected signal of the i-th band from the reference panel, and *D_i_* is the sensors’ dark current of the i-th band [8].

**Fig. 5:**
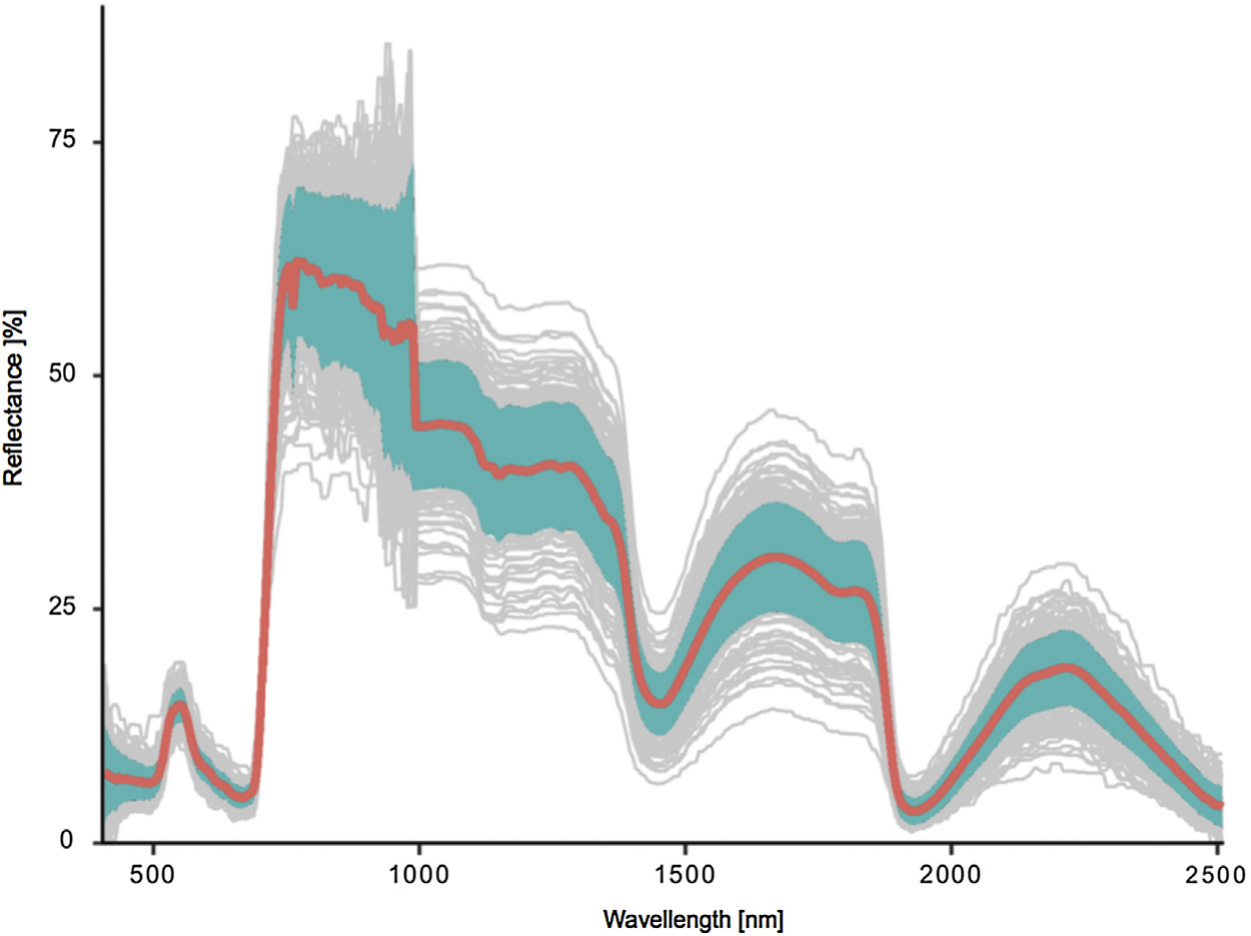
Spectral signatures of 200 randomly selected leaf-area pixels of one plant. The red line denotes mean spectrum, and the ribbon the standard deviation of the mean.

Misclassified and erroneous pixels were then removed using a set of three simple filters: (i) specular reflectance, i.e. reflectance >100% at any band, (ii) dead pixels, i.e. reflectance <0.1% at any band, and (iii) misclassifications. The latter were removed by calculating the median after removing (i) and (ii), then calculating the standard deviation around the median, and lastly, calculating the mean from the data inside 2 standard deviations around the median (Fig. 5). In contrast to median values, means incorporate spatial variability, and were hence chosen for further analysis. Spectral data extraction and filtering was repeated for each plant inside each image, separately for both VNIR and SWIR cameras. Mean values from both cameras were then combined to obtain a complete spectral signature of each plant. These data sets were then fused with the label data. Labels included imaging session (three labels), watering regime (two labels), infestation (two labels), and treatment (six labels).

Reflectance data were smoothed by Savitzky-Golay filter using second-order polynomials, and second-order derivatives were calculated to remove scattering effects in the spectra and emphasize small spectral variations not evident in the raw data. Partial least squares – discriminant analysis (PLS-DA) [14] was used as an additional pre-processing step, and to identify relevant spectral regions by evaluating their correlations with PLS-DA factors. Outliers were identified using Hotelling T^2^ test. Variables used in PLS-DA were weighted using a standard deviation weighting process, and models were validated using full cross-validation. The thus obtained PLS-factor scores were then used as input variables for support vector machine classification (PLS-SVM).

### Classification

For each PLS-SVM classification the capacity factor (C) and gamma value were determined by performing a grid search of several combinations of C and gamma on a log scale. The combinations giving the best accuracy were selected for model development. Similar to PLS-DA, variables (PLS-factors) were weighted using standard deviation weighting. Models were validated using 10-fold cross validation.

Analyses were first performed on pooled samples, from all three imaging sessions, and later separately for each imaging session. Finally, samples were analysed separately for well-watered and water-deficient plants, and infected plants alone, to determine the infection severity. Image pre-processing and data extraction were performed in ENVI 5.1 (Harris Geospatial, USA). Spectral pre-processing, PLS-SVM evaluation, and assessment of relevant spectral regions were carried out in R [15], while PLS-DA and PLS-SVM classifications were done in Unscrambler 10.3 (CAMO Software, Norway).

### Method validation

By combining hyperspectral imaging and PLS-SVM biotic and abiotic drought stress in tomato plants can be differentiated. When all imaging sessions were pooled, a 92% classification success was achieved for identification of watering regime, while determination of infested plants attained a 77% success, and treatment groups 24.6%. Time-series analysis showed that separation according to time of imaging increased classification success in all groups (watering regime, infestation, and treatment). Imaging sessions were classified with a 95% success. Their differences were characterized at spectral ranges 510–576, 606–693, 725–784, 905–909, 1047–1178, 1216–1265, 1330–1390, 1523–1553, 1830–1873, and 1906–2015 nm. Plants in drought stress showed lower reflectance in the green part of the visible spectrum and in NIR, and higher reflectance in SWIR. Determination of watering regime in time-series data achieved 100% accuracy. These differences were characterized in the NIR (831–875, 966–977, and 983–1009 nm) and SWIR (1140–1156, 1254–1270, and 1390–1400 nm) regions [16]. Changes in pigment structure are indicated by the relevance of the green and red spectral ranges. NIR and SWIR are linked to physical and chemical characteristics, such as lignin [17], as well as carbohydrates, proteins, and water content [18].

Determination of infestation showed similar patterns in all three imaging sessions. In the visible part of the spectrum no apparent differences were observed, while infested plants showed higher reflectance in NIR, and lower in SWIR. Classification success ranged from 90.5% (imaging session 2) to 100% (imaging session 1). When the data was further divided according to watering regime, classification success increased to 100% for all imaging sessions, except for water-deficient plants in session 2 (95.2% success rate).

For identification of treatment groups, the SWIR region was most relevant. Plants with low inoculum showed higher reflectance at 1286–1313, 1482–1514, 1585–1612, 1775–1835, 1982–2009, 2090–2112, and 2291–2308 nm, and lower at ranges 1395–1406, 1889–1917, and 2188–2221 nm. Furthermore, in water deficient plants, at session 2 and session 3, the NIR and visible spectral regions were relevant. Classification success was highest when data was separated according to water availability, and increased from session 1 to session 3 (from 78.6% to 100%).

Plants in drought stress reallocate resources to leaves with a higher potential, and prolonged stress leads to degradation of pigments and other structural changes [19]. Wavelengths in the ranges 966–977, 983–1009, 1216–1270, and 1330–1390 nm are linked to the O—H stretch in water, while the range 1047–1178 nm is linked to the N—H stretch of proteins [20]. Some of these spectral ranges are related to certain physiological processes, which indicate drought stress. A lower reflectance at 535 nm in linked to an increase in zeaxanthin content, which leads to the photoprotective state of the zeaxanthin cycle [21]. On the other hand, a decrease at 550 nm is related to adjustments of photoprotective pigments, such as anthocyanins [22]. As plants matured and RKN infestations became more established, the relevant spectral ranges began shifting toward the visible spectrum (from S1 to S3). In the last imaging session all plants in stress showed visible signs, mostly in the green part of the spectrum (511–566 nm). The damage due to stress became severe enough to overshadow foliar water content (at 1390–1520 and 1860–2080 nm). Moreover, the O—H stretch was replaced by the C—H stretch of carbohydrates and proteins (at 1189–1222 and 1324–1346 nm) for the identification of infestation and its severity. Hence, wavelengths linked to pigments, and leaf chemistry and structure are important for the determination and assessment of infestations.

### Novelty of the methodology

Different methods enable early detection of stress in plants, but lack the potential to identify a specific disease [23]. Identification of RKN infestations is further complicated by the similarity of visual symptoms with those of drought stress. Individually, drought stress and RKN infestations have been detected before using hyperspectral spectrometers [24,25], with varying degrees of success. Broad-band vegetation indices were developed for multispectral sensors, and are an easy and quick way of analyzing data, and can provide useful information. But they employ only certain wavelengths, thus ignoring most of the data hyperspectral system provide. For example, an easy screening method for drought stress is reflectance at the water absorption band at 950–970 nm [26]. But our results indicate that this changes depending on plant development and infestation status [16]. Furthermore, as plants mature and infestations become more established, relevant spectral ranges can shift. Such a change cannot be detected by using vegetation indices, since these are linked to certain wavelengths.

Our method has several benefits, which increase classification accuracy: (1) imaging sensors provide more data, from the entire canopy, unlike spectrometers where individual measurements of small leaf are need to be made to obtain enough data; (2) we utilize the light spectrum from 400 to 2500 nm (i.e. VNIR and SWIR); and (3) we do not calculate spectral indices, thus limiting the available information to just a few selected wavelengths, but instead consider all wavelengths. The SWIR spectral region is of special importance, as it’s linked to physical and chemical leaf characteristics, such as cellulose and lignin [27], and carbohydrates, proteins, and water content [28].

Novelty of the presented methodology is that with the use of hyperspectral imaging detection of root-knot nematode (*Meloidogyne* spp.) infestation and its differentiation from water deficiency is possible. Moreover, hyperspectral imaging enables early detection of infestation, prior to development of visible signs, thus facilitating timely management practices. There is no other reliable method to determine nematode infestation without physical examination of plant roots, which is a disruptive method as the plant needs to be removed from the soil. This remote sensing approach could be very valuable for producers as it is discussed in more detail in Susič et al. [16].
